# Carbapenem-resistant *Acinetobacter baumannii*: A challenge in the intensive care unit

**DOI:** 10.3389/fmicb.2022.1045206

**Published:** 2022-11-10

**Authors:** Yuan Jiang, Yinhuan Ding, Yueshuai Wei, Chunxia Jian, Jinbo Liu, Zhangrui Zeng

**Affiliations:** Department of Laboratory Medicine, The Affiliated Hospital of Southwest Medical University, Luzhou, China

**Keywords:** carbapenem-resistant *Acinetobacter baumannii*, intensive care unit, infection, environmental contamination, resistance mechanism, homology, treatment and control strategies

## Abstract

Carbapenem-resistant *Acinetobacter baumannii* (CRAB) has become one of the leading causes of healthcare-associated infections globally, particularly in intensive care units (ICUs). Cross-transmission of microorganisms between patients and the hospital environment may play a crucial role in ICU-acquired CRAB colonization and infection. The control and treatment of CRAB infection in ICUs have been recognized as a global challenge because of its multiple-drug resistance. The main concern is that CRAB infections can be disastrous for ICU patients if currently existing limited therapeutic alternatives fail in the future. Therefore, the colonization, infection, transmission, and resistance mechanisms of CRAB in ICUs need to be systematically studied. To provide a basis for prevention and control countermeasures for CRAB infection in ICUs, we present an overview of research on CRAB in ICUs, summarize clinical infections and environmental reservoirs, discuss the drug resistance mechanism and homology of CRAB in ICUs, and evaluate contemporary treatment and control strategies.

## Introduction

The incidence of drug-resistant organism infection is currently increasing in hospitals and other clinical care settings, particularly in ICUs. A place that provides life support for critically ill or unconscious patients, ICU is the cornerstone of life extension for critically ill patients. However, because of a delayed immune response, reduced host defense, and use of invasive devices—central venous catheterizations, mechanical ventilation, and urinary tract catheterizations—patients in ICUs have an increased risk of infection. The morbidity and mortality of such infections have been reduced by the extensive use of antibiotics in recent decades. However, with the rise of the use of antibiotics in the treatment of microbial infections, increased selection pressures promote the emergence and dissemination of drug-resistant pathogens (Lipsitch and Samore, 2002; Esposito and Leone, 2007; Baker et al., 2018).

A significant positive association between antibiotic resistance rates and antibiotic consumption has been determined, together with a rising trend in antimicrobial resistance (Agodi et al., 2015). Carbapenems were once recognized as a pillar of treatment for clinical critical infections, but with their widespread use, resistance to carbapenems has increased as well. The emergence and dissemination of carbapenem-resistant non-fermenting Gram-negative bacilli (NFGNB) in ICUs pose a substantial threat in hospitals (Agarwal et al., 2017; Kousouli et al., 2019). Among these bacteria, CRAB is increasingly becoming one of the leading causes of healthcare-associated infections (HAIs), particularly in ICUs (Blanco et al., 2018; Busani et al., 2019; Chen et al., 2019; Tomczyk et al., 2019). In the *Global Priority List of Antibiotic-Resistant Bacteria* published by the World Health Organization (WHO) in 2017, CRAB was classified as among those bacteria for which antibiotics are most urgently needed (Tacconelli et al., 2018).

CRAB has been associated primarily with respiratory tract infections in ICUs, particularly ventilator-associated pneumonia (VAP; Nhu et al., 2014; Karruli et al., 2021; Khalil et al., 2021; Said et al., 2021; Pogue et al., 2022). Although no definitive agreement has been reached on the links between CRAB infections and an increased risk of mortality, CRAB infections have exhibited a significant association with the length of ICU stay, increased patient costs, and antibiotic use (Phu et al., 2017; Kousouli et al., 2019; Zhen et al., 2020; Liu Y. et al., 2020; Ejaz et al., 2021). Polymyxin currently remains effective as a treatment method for CRAB infections in ICUs (Garnacho-Montero et al., 2015; Sana et al., 2021). However, on an individual-patient basis, the use of polymyxin remains rather limited because of nephrotoxicity and neurotoxicity (Nazer et al., 2015b; Katip and Oberdorfer, 2021; Liu et al., 2021; Zhang N. et al., 2021). The emergence of polymyxin resistance in *A. baumannii* has also been reported (Cheah et al., 2016a,b; Carrasco et al., 2021). Under these conditions, the control and treatment of CRAB in ICUs can potentially face new challenges and have prompted growing concern in the medical community.

Therefore, evidence-based interventions to strengthen prevention and control initiatives are urgently needed. Funding, research, and development of new antimicrobials should pay increased attention to CRAB infections in ICUs. This review focuses on CRAB infections in ICUs and its transmission, mechanisms of resistance, treatment alternatives, and control strategies to provide a basis for prevention and control countermeasures for CRAB infections in ICUs.

## CRAB in ICUs

### CRAB infections of patients in ICUs

WHO estimates that about 30% of ICU patients are affected by at least one HAI in high-income countries; meanwhile, the frequency is at least two-fold to three-fold higher in middle- and low-income countries (World Health Organization, 2011). NFGNB is the leading cause of HAI, among which *A. baumannii* is an opportunistic pathogen that causes hospital-acquired septicemia, pneumonia, and urinary tract infections, particularly in ICUs (Antunes et al., 2014; Harding et al., 2018). Notably, as CRAB isolates in growing numbers have been isolated from patients, the prevalence and risk factors of CRAB infections have received increasing attention.

VAP, a severe complication, remains to be the most common infection acquired in ICUs (Kalanuria et al., 2014; Kalil et al., 2016). The pathogens responsible for VAP and their resistance mechanisms in ICUs are difficult to identify. The emergence and popularity of CRAB, which causes pulmonary infection in ICUs, have been reported in numerous publications. One multicenter prospective study found that multidrug-resistant Gram-negative bacteria, including *A. baumannii*, *K. pneumoniae*, and *P. aeruginosa*, are frequently associated with VAP in ICUs (Bandić-Pavlović et al., 2020). In a study conducted over a period of 46 months, Lambiase et al. demonstrated that *A. baumannii* isolated from patients with VAP in ICUs were resistant to carbapenem with imipenem MIC ≥ 16 μg/ml (Lambiase et al., 2012).

A retrospective study further found that the sputum separation rate of CRAB from ICUs was markedly higher than those from non-ICUs, and the resistance rate of CRAB showed a significantly rising trend (He et al., 2020). Similarly, 80% of CRAB in ICUs were isolated from sputum specimens, and CRAB comprised more than 50% of carbapenem-resistant Gram-negative bacilli (Karuniawati et al., 2013; Lăzureanu et al., 2016). Alternatively, another study showed that 58 of 61 *A. baumannii* isolates exhibited MICs with imipenem or meropenem≥16 μg/ml, and pulmonary infection was the most common site (26 of 36 cases; Mammina et al., 2012). As is widely known, the use of mechanical ventilation is strongly associated with the incidence of VAP. Therefore, when lung infection due to CRAB occurs in ICUs, the use of ventilators should be paid more attention than other wards to prevent cross-infection. However, beyond the use of mechanical ventilation, independent risk factors for CRAB causing pulmonary infections have been identified, such as previous stays in other departments, longer ICU stay, and previous use of carbapenems (Nazer et al., 2015a; Djordjevic et al., 2016). That is to say, a comprehensive infection control strategy is required to effectively control the emergence and spread of CRAB in ICUs.

Zhou et al. reported that the high mortality associated with bloodstream infections (BSIs) caused by *A. baumannii* has become a major clinical concern (Zhou et al., 2019). As such, increased attention should be paid to patients with CRAB bacteremia, apart from those with pulmonary CRAB infections. Invasive procedures and excessive use of antibiotics, particularly in patients with compromised immunity, are risk factors independently correlated with CRAB bacteremia (Kim et al., 2012; Shirmohammadlou et al., 2018). Previous studies have also found that colonization in the respiratory tract and gastrointestinal tract by CRAB is a crucial step before nosocomial infection (Lazareva et al., 2014; Bado et al., 2018; Kiddee et al., 2018; Maamar et al., 2018). Identifying risk factors and providing targeted interventions may become effective approaches to reducing the incidence of CRAB-causing HAIs.

### Environmental contamination of CRAB in ICUs

*A. baumannii* can persist in the environment for long-term periods. *A. baumannii,* which ubiquitously and continuously persists in the hospital setting is one of the main sources of HAIs (Sunenshine et al., 2007). Ng et al. reported that environmental CRAB contamination was detected in nearly two-thirds of the rooms housing patients with CRAB (Ng et al., 2018). Previous studies have also indicated that cross-contamination of multidrug-resistant bacteria, specifically CRAB in ICUs, may occur *via* the air (Shimose et al., 2016), high-density electroencephalogram material (Weiss et al., 2016), Velcro on blood pressure cuffs (Alfandari et al., 2014), medical devices, furniture, and gloves (Raro et al., 2017). Uwingabiye et al. also observed genetic similarity between environmental and clinical CRAB isolates in 96.4% of all isolates (Uwingabiye et al., 2017). Given the intersection between patients and the environment, increasing studies have been devoted to the study of the extensive environmental colonization of CRAB in ICUs.

CRAB from environmental (environment and healthcare workers from ICUs) and clinical samples has been isolated and analyzed in several studies (Royer et al., 2015; Raro et al., 2017; Jain et al., 2019; Al-Hamad et al., 2020; Liu W. et al., 2020; Wang et al., 2021), and it was found that the overwhelming majority of CRAB isolated from clinical and environmental samples produced OXA-23, but no clone was specifically responsible for both environmental colonization and ICU infections. However, the experimental results of pulsed-field gel electrophoresis (PFGE) have revealed the spread of carbapenem-resistant isolates via cross-transmission among the environment and patients (Royer et al., 2015; Jain et al., 2019). Similarly, Salehi et al. (2018) and Raro et al. (2017) analyzed the isolates of *A. baumannii* from patients, staff, and the environment, and emphasized the circulation of CRAB as a nosocomial pathogen in different wards of hospitals, particularly in ICUs. Shenoy et al. also identified highly contaminated areas and confirmed the role of environmental reservoirs by investigating five clinical CRAB infection cases (Shenoy et al., 2020). It should be noted that although environmental contamination of CRAB in ICUs has gained increasing attention, research about whether specific clone was responsible for both environmental colonization and ICU infections is still lacking.

Further, a prospective surveillance study for 8 months has shown that more than half of the CRAB strains originating from the air are clonally associated with the clinical strains of nine patients in two medical ICUs with 20 beds total (Yakupogullari et al., 2016). The results of this study (Yakupogullari et al., 2016) suggested that infected patients can spread CRAB in large quantities to the air of an ICU, and these strains can still infect new patients after several months. This conclusion was verified by another study in China (Jiang et al., 2018). That is to say, special infection control measures may be required to prevent the airborne spread of CRAB in ICUs.

Colonization is usually regarded as a fundamental ecological process. Bacterial colonization of the surfaces is almost ubiquitous, especially in healthcare settings. In addition, the colonization of gastrointestinal tract, respiratory tract, urinary tract, and axilla is also receiving growing interest from researchers. Previous studies have found that colonization is an essential step in pathogen infections (Chipolombwe et al., 2016; Weiser et al., 2018; Tanimoto et al., 2019). Besides, numerous studies have shown that colonization is an important risk factor for subsequent infection (Huang et al., 2006; Haber et al., 2007; Martin et al., 2016; Gorrie et al., 2017). Studying the relationship between colonization and infections may bring new insights into disease prevention and treatment.

**Figure 1:**
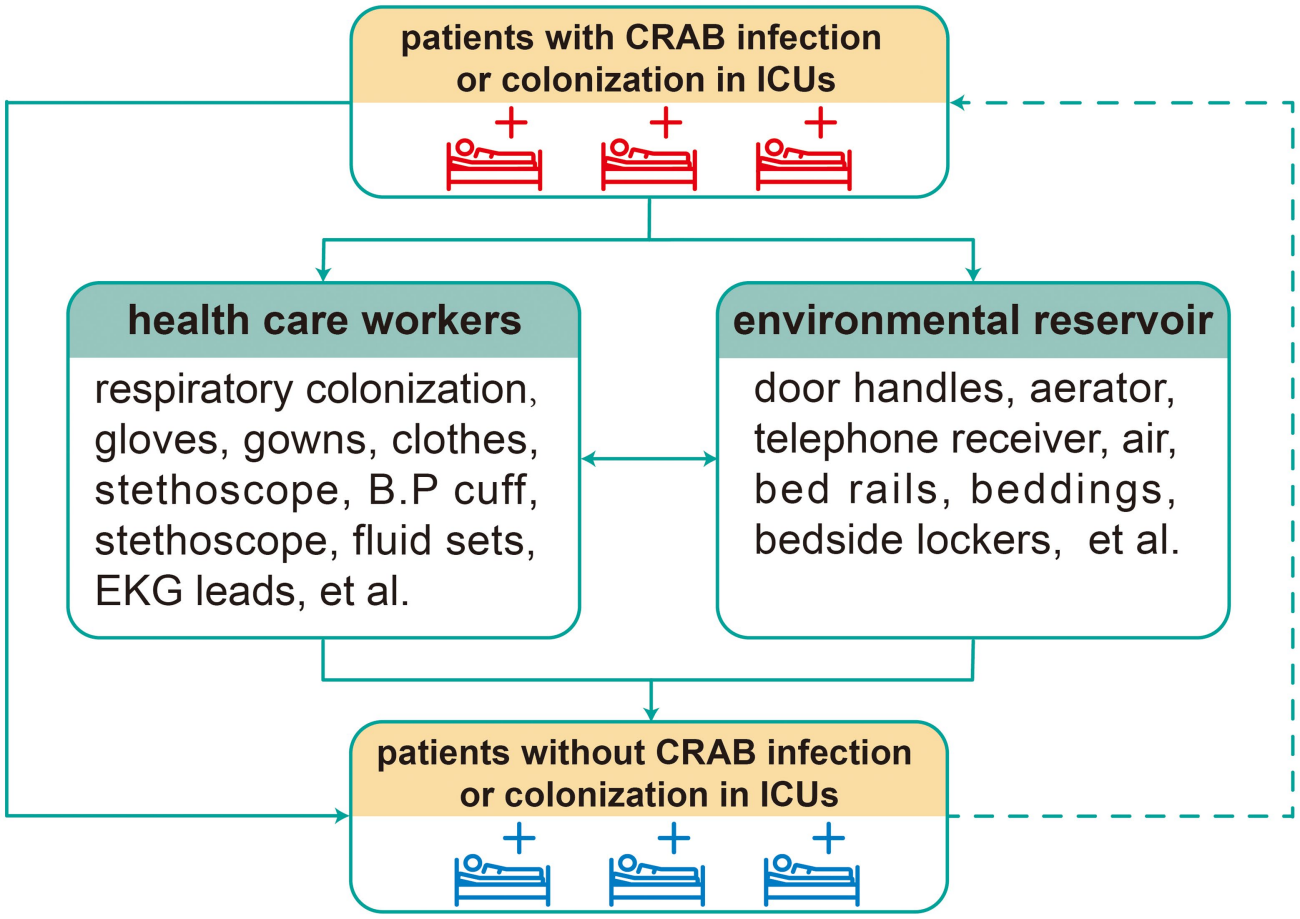
Transmission relationship of CRAB in ICUs. Solid arrow: bacterial infection and dissemination; dotted arrow: patients recently infected and colonized with CRAB are viewed as the new transmission source.

Interestingly, several studies have found that air and environmental surface contamination of CRAB were significantly greater among patients with respiratory tract colonization and gastrointestinal colonization than in other types of patients (Rosa et al., 2014; Shimose et al., 2016). Moreover, in a retrospective cohort study, significant associations were observed between CRAB colonization and clinical infections (Latibeaudiere et al., 2015). Namely, respiratory tract colonization and gastrointestinal colonization also play prominent roles in CRAB infections in ICU patients. Meanwhile, the relationship between environmental contamination and pathogen infections also cannot be neglected in general wards (Al-Hamad et al., 2020). Overall, environmental reservoirs of CRAB play a pivotal role in the HAIs of CRAB. An intensive study of environmental CRAB is beneficial to the control and elimination of CRAB infections in ICUs. The figure below shows the transmission relationship of CRAB among patients, health care workers, and the environment in ICUs (Figure 1).

## Drug resistance mechanism and homology of CRAB isolated from ICUs

The increase in drug-resistant bacterial infections leads to a heavy burden on healthcare systems globally. The study of drug resistance mechanisms is the first step to overcoming the infection of drug-resistant bacteria. CRAB has been classified by the WHO as one of the 12 top priority resistant bacteria presenting the most serious threat to public health (Tacconelli et al., 2018). As described previously, CRAB has been a major cause of HAIs (Sunenshine et al., 2007). Accordingly, a significant understanding of the mechanism underlying CRAB resistance is of major importance for drug development and clinical therapy.

An increasing number of researchers have conducted studies on drug resistance mechanisms and the homology of CRAB isolated from ICUs (Table 1). Details of the Table 1 reveal that most studies on the molecular characterization of CRAB in ICUs have been conducted in Asia and Africa, primarily in the developing world. Although the antibiotic resistance genes (ARGs) in the majority of studies have been dominated by *bla*_OXA-23-like_, differences in ARGs and CRAB molecular typing have been found between different countries and regions.

**Table 1:** Studies on CRAB and its ARGs isolated from ICU patients.

Country	References	Period of study	Number of CRAB	Wards	ARGs	Molecular Typing
Tunisia	Maamar et al. (2018)	Dec 2014 to Feb 2015	13	ICU	*bla*_OXA-23_ (84.6%), *bla*_NDM-1_ (15.4%), *armA* (84.6%), *tetB* (84.6%), *sul1* (84.6%), *catB* (84.6%), *aph(3′)-VIa* (69.2%), *aph(3′)-Ia* (15.4%), *ant(2′)-Ia* (15.4%), *bla*_TEM-1_ (30.8%)	ST195 (84.6%), ST1089 (15.4%)
Egypt	Ramadan et al. (2018)	Jul 2017 to Dec 2017	30	ICU	*bla*_OXA-23_ (90%), *bla*_NDM_ (66.7%), *bla*_GES_ (50%)	Unknown
Uganda	Aruhomukama et al. (2019)	Jan 2015 to Dec 2017	21	ICU (16)	*bla*_VIM_ (100%), *bla*_VIM_ + class 1 integron (61.9%), *bla*_OXA-23_ (29%), *bla*_OXA-24_ (24%), *bla*_OXA-51_ (100%)	Unknown
				Other wards (5)		
Egypt	Khalil et al. (2021)	May 2019 to Feb 2021	54	ICU	*bla*_OXA-23-like_ (88.9%), *bla*_NDM_ (27.7%), *bla*_OXA-51_ (9.2%), *Bap* (25.9%), *bla*_PER-1_ (11.1%)	REP-PCR Genotyping: four distinctive REP-PCR clusters (A-D) and two (746A, 715A) singleton isolates
Nigeria	Odih et al. (2022)	Aug 2017 to Jun 2018	34	ICU	*bla*_OXA-51-like_ (100%), *bla*_OXA-23-like_ (50%), *bla*_NDM-1_ (44.1%), *bla*_OXA-420_ (5.9%), *bla*_TEM-84_ (29.4%), *sul1* (29.4%), *sul2* (29.4%)	ST2 (29.4%), ST85 (23.5%), ST149 (8.8%), ST25 (5.8%), ST164 (5.9%), other STs (17.6%)
China	Zhao et al. (2019)	Jan 2010 to Dec 2017	21	ICU	*bla*_OXA-23_ (100%), *bla*_OXA-24_ (28.6%), *bla*_OXA-51_ (100%), *bla*_ADC_ (100%), *bla*_TEM_ (95.2%), IS*Abal* (95.2%), IS*A-23* (95.2%), IS*A-ADC* (28.6%)	ST2 (95.2%)
						ST1119 (4.8%)
China	Qian et al. (2015)	Jan 2010 to Jan 2014	140	ICU (48)	*bla*_OXA-23_ (81.3%), *bla*_OXA-24_ (5.3%), *bla*_OXA-51_ (61.3%), *imp* (12.3%), *intl1* (57.9%), *qacE*Δ*1-sul1* (61.2%)	ST208 (52.1%)
				Respiration Medicine (49)		ST218 (47.9%)
				Burn and Plastic Surgery (43)		
China	Zhang et al. (2021c)	Jul 2018 to Jun 2019	91	ICU	*bla*_OXA-51-like_ (100%), *bla*_OXA-23-like_ (93.4%), *bla*_OXA-24-like_ (2.2%), *bla*_OXA-58-like_ (2.2%), IS*Aba1*/*bla*_OXA-51-like_ (27.5%), *bla*_NDM-1_ (8.8%)	Unknown
China	Wang et al. (2021)	Jul 2017 to Dec 2017	61	ICU	*bla*_OXA-51-like_ (100%), *bla*_OXA-23-like_ (100%), *bla*_AmpC_ (100%), IS*Aba1* (100%), *gyrA* mutation (95.1%)	ST208 (93.5%), ST369 (1.6%), ST373 (4.9%)
China	Zhang et al. (2021b)	Jan 2013 to Dec 2018	105	ICU	*bla*_OXA-23_ (100%), *bla*_OXA-66_ (100%), *bla*_ADC-25_ (100%), *bla*_TEM-1D_ (81.9%), *aac(6′)-Ib* (15.2%), *aac(6’)Ib-cr* (15.2%), *aph(3′)-Ia* (16.2%), *aph(3′)-Ib* (81.0%), *aph(6′)-Id* (83.8%), *armA* (97.1%), *aadA* (83.8%), *mph(E)* (97.1%), *msr(E)* (97.1%), *catB8* (15.2%), *tet(B)* (83.8%), *sul1* (16.2%), *sul2* (44.8%)	ST2 (100%)
China	Chen et al. (2018b)	Jan 2014 to Dec 2016	78	ICU (51)	*bla*_OXA-51-like_ (100%), *bla*_OXA-72_ (57.7%), *bla*_OXA-23-like_ (42.3%), *bla*_OXA-58-like_ (1.3%)	ST2 (100%)
				Other wards (27)		
China	Zhou et al. (2015)	May 2012 to Nov 2013	46	ICU	*bla*_OXA-51_ (100%), *bla*_OXA-23_ (100%), *bla*_OXA-51-like_ + IS*Aba1-bla*_OXA-23-like_ (84.8%)	ST195 (54.4%), ST365 (19.3%), ST92 (8.8%), ST381 (5.3%), ST75 (1.8%), five novel ST isolates (10.7%)
China	Liu and Liu (2021)	Jul 2019 to Jan 2020	60	ICU (30)	*bla*_OXA-23_ (80%), *bla*_VIM-2_ (23.3%), *bla*_IMP-4_ (40%), *bla*_NDM-1_ (20%), *ampC* (16.7%), mutation of *CarO* (86.7%)	ST92 (63.3%), ST111 (20.0%), ST244 (10.0%), ST357 (6.7%)
				Respiratory		
				Department (30)		
China	Guo and Li (2019)	Jan 2017 to Jan 2018	82	ICU	*bla*_OXA-51_ (100%), *bla*_OXA-23_ (100%), *qac*Δ*E1* (76.8%), *qacE* (30.5%)	ST540 (36.6%), ST195 (22.0%), ST208 (18.3%), ST191 (13.4%), ST369 (4.9%), ST469 (2.4%), ST381 (1.2%), ST136 (1.2%)
Pakistan	Ejaz et al. (2021)	Sep 2020 to Dec 2020	113	ICU (81)	*bla*_OXA-51_ (100%), *bla*_OXA-23_ (49.5%), *bla*_NDM-1_ (24.7%), *bla*_OXA-58_ (19.4%), *bla*_OXA-143_ (2.6%)	ST2 (46.9%), ST1 (15.9%), ST589 (12.3%), ST7 (9.7%), ST158 (8.8%), ST23 (4.4%), ST25 (1.7%)
				Other wards (32)		
Iran	Shirmohammadlou et al. (2018)	Jun 2014 to Mar 2016	100	ICU	*bla*_OXA-23_ (89%), *bla*_OXA-24_ (29%), *bla*_OXA-51_ (100%), *bla*_VIM_ (8%), *qac*Δ*E1* (91%), *qacG* (10%), *qacE* (4%)	unknown
Iran	Salehi et al. (2019)	Aug 2016 to Feb 2017	180	ICU (134)	*bla*_OXA-23_ (60.5%), *bla*_OXA-58_ (17.2%), *bla*_OXA-24_ (1.7%)	Unknown
				Other wards (46)		
Thailand	Kiddee et al. (2018)	Dec 2014 to Dec 2015	43	ICU	*bla*_IMP_ (2.3%), *bla*_NDM_ (2.3%), *bla*_OXA-23_ (4.7%), *bla*_OXA-24_ (2.3%), *bla*_OXA-51_ (14.0%), *bla*_OXA-23/51_ (46.5%), *bla*_OXA-51/58_ (9.3%), *bla*_OXA-23/51/58_ (11.6%), *bla*_OXA-24/51/58_ (2.3%)	Unknown
Croatia	Bandić-Pavlović et al. (2020)	Sep 2017 to Mar 2018	23	ICU	*AmpC* (100%), *bla*_OXA-23_ (34.8%), *bla*_OXA-24_ (52.2%)	ST1 (13.0%), ST2 (87.0%)
Italy	Mammina et al. (2012)	Oct 2010 to Mar 2011	61	ICU	*bla*_OXA-51-like_ (100%), *bla*_OXA-23_ (80.3%), *bla*_OXA-58_ (3.3%)	ST2 (96.7%), ST78 (3.3%)
Italy	Mezzatesta et al. (2014)	Jan 2013 to Jul 2013	52	ICU	*bla*_OXA-51_ (100%), *bla*_OXA-23_ (100%)	ST2 (100%)
Italy	Venditti et al. (2019)	Dec 2016 to Apr 2017	13	ICU (12)	*bla*_OXA-51-like_ (100%), *bla*_OXA-23_ (100%)	ST2 (100%)
				Other wards (1)		
Brazil	Neves et al. (2016)	Dec 2009 to Dec 2010	56	ICU	*bla*_OXA-51_ (100%), *bla*_OXA-23_ (51.2%), *bla*_OXA-143_ (18.6%)	Unknown
Uruguay	Bado et al. (2018)	Aug 2010 to Jul 2011	78	ICU	*bla*_OXA-51_ (100%), *bla*_OXA-23_ (79.5%), *bla*_OXA-58_ (3.8%)	ST79 (95.5%), ST958 (4.5%)

These literature reviews reflect the high diversity of the ARGs of CRAB isolated from ICUs globally. Zhang X. et al. (2021) investigated the phylogenetic relationships of 105 CRAB isolates from an ICU of a Chinese hospital and found that CRAB isolates contained 17 unique ARGs. And whole-genome sequencing (WGS) revealed the presence of *bla*_ADC-25_, *bla*_OXA-23_, and *bla*_OXA-66_ in all strains, which belonged to Sequence type (ST) 2 (Zhang X. et al., 2021). A previous study indicated that carbapenem resistance was dominantly driven by the dissemination of CRAB isolates carrying *bla*_OXA-23_, belonging to ST2 (Mammina et al., 2012). A study conducted in Pakistan also found that the ST2 clone-harboring *bla*_NDM-1_ and *bla*_OXA-23_, which are widely distributed in ICUs, could prompt increased mortality (Ejaz et al., 2021). Notably, a study in another Chinese hospital in Shanghai reported the presence of *bla*_OXA-23_ in all CRAB strains (Wang et al., 2021), and it was found that the predominant clone of CRAB was ST208, which was consistent with the results obtained by Qian et al. (2015).

Unlike these studies, Zhang et al. found patterns of *bla*_OXA-23_ (93.4%), IS*Aba1*/*bla*_OXA-51-like_ (27.5%), *bla*_OXA-24_ (2.2%), *bla*_OXA-58_ (2.2%), and *bla*_NDM-1_ (8.8%) in CRAB strains (Zhang Y. et al., 2021). On the basis of their results (Zhang Y. et al., 2021), the IS*Aba1*/*bla*_OXA-51-like_ and *bla*_OXA-23-like_ might be more relevant to resistance in CRAB. Although the ARGs in CRAB varied in different regions, *bla*_OXA-23_ was always the principal ARG in CRAB (Mahamat et al., 2016; Neves et al., 2016; Salehi et al., 2019). The *bla*_OXA-23_ is mostly found on plasmids, and Corvec et al. (2007) found that IS*Aba1* and IS*Aba4* which were detected upstream of the *bla*_OXA-23_ gene, provided promoter sequences for its expression.

In addition to *bla*_OXA-23_, ARGs such as *bla*_OXA-24/40_, *bla*_OXA-51_, *bla*_OXA-58_, and *bla*_OXA-72_ also play prominent roles in the drug resistance of CRAB (Schultz et al., 2016; Mavroidi et al., 2017; Chen et al., 2018b; Salehi et al., 2019; Zhang Y. et al., 2021). The *bla*_OXA-24-like_ genes have been identified as chromosomally encoded. And Azizi et al. found that the isolates of *A. baumannii* with both *bla*_OXA-23_ and *bla*_OXA-24_ had strong biofilm-forming capability (Azizi et al., 2015). It is well known that biofilms can facilitate the development of antibiotic resistance by limiting bacterial exposure to antibiotics. Apart from the aforementioned genes, biofilms and AdeABC efflux-pump genes have also been detected in CRAB isolated from ICUs (Mavroidi et al., 2017; Chen et al., 2018a; Khalil et al., 2021). However, there are relatively few studies on biofilms and efflux-pump of CRAB in ICUs. It seems highly likely that the biofilms and efflux-pump of CRAB have not gained enough attention in ICUs.

The emergence of CRAB may be promoted by the adaptation and dissemination of a diverse group of successful clones. In a retrospective study of the drug resistance and distribution of pathogens isolated from the ICUs of 12 hospitals, Liu et al. found homology in the PFGE typing of CRAB (Liu W. et al., 2020). Moreover, Salehi et al. demonstrated that nine cross-existing clones consisting of eight cluster types and one ST were present between two hospitals (Salehi et al., 2019). Likewise, in South Africa, a study involving two hospitals found that ST106, ST229, ST258, and ST208 were established in both hospitals; meanwhile, ST339, ST502, and the novel ST1552 were established in Hospital B only, whereas ST848 was established in Hospital A only (Lowe et al., 2018). ST2 was identified as the most predominant isolate in Italy in several studies (Mammina et al., 2012; Mezzatesta et al., 2014; Schultz et al., 2016). It seems quite different from the distribution in other areas. In addition, resistance mechanisms and molecular epidemiology in the CRAB isolates varied between the general wards and ICUs. This result is supported by the PCR detection results of resistance genes, PFGE, and multilocus sequence typing (MLST) analysis in a recent study (Liu and Liu, 2021). The diverse ST types in different countries and regions have been listed in Table 1. Although CRAB infections have been reported all over the world, the lack of reports of molecular characterization targeted CRAB in ICUs is noticeable in some regions. Thus, further studies are needed to provide further evidence.

Overall, despite a certain degree of homology in CRAB from different ICUs, a high genetic diversity could not be overlooked. Further studies and investigations on the homology and drug resistance mechanism of CRAB in ICUs have important implications for reversing and reducing drug resistance, as well as developing control and treatment strategies.

## Strategies and advice

### Treatment strategies for CRAB infections

#### Guidelines for the treatment of CRAB infections

**Figure 2:**
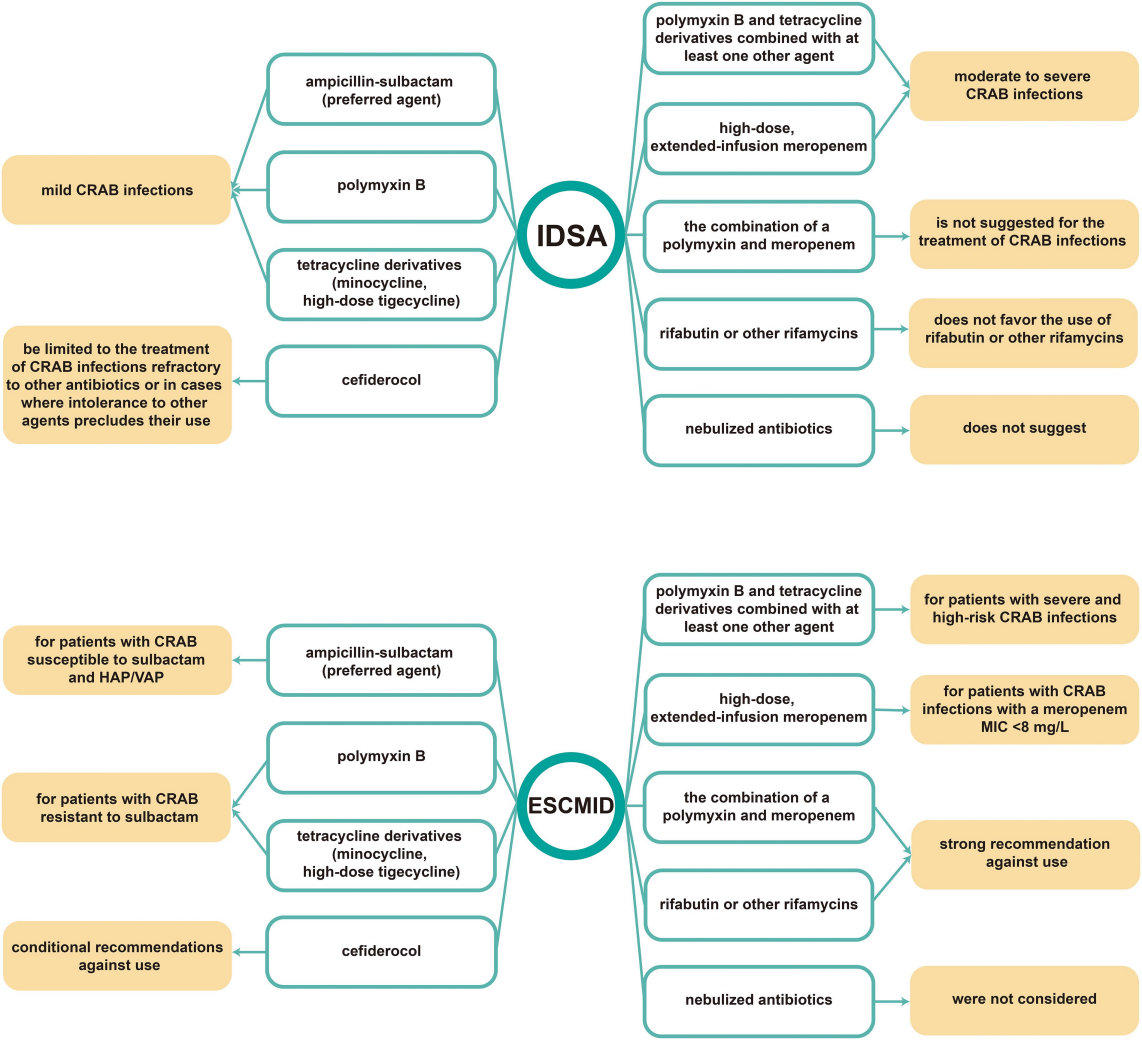
Treatment guidelines of CRAB infections for 2022 IDSA guidance and 2021 ESCMID guidance. IDSA: Infectious Diseases Society of America, ESCMID: European Society of Clinical Microbiology and Infectious Diseases.

As previously mentioned, CRAB infections have become increasingly prevalent worldwide. The increasing healthcare burden caused by CRAB in ICUs has directed widespread attention to the treatment and control of CRAB. However, limited therapeutic options, as well as the long study period and task difficulty involved in new drug development, have prompted the increased interest among researchers devoting themselves to evaluating and improving treatment regimens on the basis of existing drugs. As clinical treatment options continued to be evaluated and studied, the European Society of Clinical Microbiology and Infectious Diseases (ESCMID) set guidelines in 2021 for the treatment of multidrug-resistant Gram-negative bacilli infections (Paul et al., 2022). The Infectious Diseases Society of America (IDSA) also recently updated the guidance document on the treatment of AmpC β-lactamase-producing Enterobacterales, CRAB, and infections caused by *Stenotrophomonas maltophilia* (Tamma et al., 2022). Both guidelines include therapeutic choices for CRAB infections. Subtle differences between these two guidance documents are observed despite the similarity of the majority of treatment strategies for CRAB. The current study presents a summary in Figure 2.

In addition to the two guidelines by IDSA and ESCMID, similar guidelines including the treatment strategies related to CRAB infections have also been published in other regions and countries, such as China, Italy, and Arab countries of the Middle East (Guan et al., 2016; Al Salman et al., 2020; Tiseo et al., 2022). These recommendations present the distribution of diverse social and healthcare structures of different regions, as well as compensates for the deficiencies of international consensus guidelines. It is worth noting that, Table 1 lists China, Italy, and Arab countries of the Middle East as the main regions reporting CRAB infections in ICUs. In the consensus statement in Arab countries of the Middle East, *A. baumannii* infections are divided into two parts: (1) bacteremia and nosocomial pneumonia; (2) complicated urinary tract infection, and complicated skin and soft tissue infection. The treatment schemes, including first-choice therapy and duration, can vary based on the type of infection (Al Salman et al., 2020). In addition, three recommendations are presented in the guidance document in Italy with the following brief overview: (1) Consultation with specialists is recommended; (2) Rigorous monitoring of renal function is strongly recommended when colistin is administered; (3) Although cefiderocol represents a high-potential alternative for patients with CRAB infections, further studies need to be conducted to estimate the use of cefiderocol (Tiseo et al., 2022). In 2016, the Chinese consensus statement on the antimicrobial treatment of extensively drug-resistant Gram-negative bacilli (XDR-GNB) infections was released (Guan et al., 2016). This statement included the treatment strategies of XDR-GNB and was regarded as a reference for the treatment of CRAB infections. In 2019, the recommendations for antimicrobial treatment of CRAB infections were explicitly proposed in “*Technical Guidelines for Prevention and Control of Carbapenem-resistant Gram-Negative Bacilli Infection in China*” (Hu, 2019). An overview of these three guidelines is presented in Table 2 for a more intuitive comparison. The Table 2 shows that compared with the guidelines in Arab countries of the Middle East and China, the guidance document in Italy contains no detailed treatment strategies. Medical institutions in Italy were likely to comply with the guideline-recommended treatment strategies in ESCMID.

**Table 2:** Overview of the recommendations for antimicrobial treatment of CRAB infections in Arab countries in the Middle East, Italy, and China.

Arab countries of the Middle East		Italy	China
**Bacteraemia and nosocomial pneumonia:** **stable patients:** Colistin^a^ or polymyxin B monotherapy critically ill patients: Colistin^a^ or polymyxin B in combination with one of the following: Aminoglycoside^b^ Ampicillin/sulbactam Carbapenem (high-dose, extended infusion) Fosfomycin Minocycline Rifampicin Tigecycline (high-dose)	**cUTI and cSSTI** Monotherapy with one of the following: Aminoglycoside Ampicillin/sulbactam Carbapenem (high-dose, extended infusion) Colistin Doxycycline^c^ Fosfomycin^d^ Tigecycline^c^ Trimethoprim-sulfamethoxazole	Strongly recommend a consultation by specialists; Rigorous monitoring of renal function is strongly recommend when colistin is administered; Further studies are necessary to estimate the use of cefiderocol.	**First choice according to drug sensitivity results:** Compound preparation containing sulbactam Aminoglycoside Fluoroquinolone Minocycline SMZ-TMP **Secondary choice according to drug sensitivity results:** Tigecycline Polymyxin Other β Lactamase inhibitor compound preparations

cUTI, complicated urinary tract infection; cSSTI, complicated skin and soft tissue infection; SMZ-TMP, sulfamethoxazole-trimethoprim. ^a^ intravenous and inhaled (nebulized) for patients with pneumonia. ^b^ for bacteraemia only. ^c^ for cSSTI only. ^d^ for cUTI only.

In summary, although many regions have proposed treatment guidelines and consensus on CRAB, the recommendations for the antimicrobial treatment of CRAB infections differed widely among different areas on the basis of the variations in antimicrobial availability, local preferences, and resistance patterns. Moreover, these recommendations need to be updated periodically in accordance to the evolution and spread of antibiotic resistance, and the advent of novel therapeutic strategies.

#### Other studies for treatment strategies

Despite the growing availability of guidelines, numerous questions about the treatment of CRAB infections arise. Ehrentraut et al. found that the use of colistin without drug concentration monitoring might be unsafe for critically ill patients, and treatment in accordance with guidelines does not ensure efficient target levels (Ehrentraut et al., 2020). The high frequency of isolation of CRAB in ICUs requires accurate antimicrobial susceptibility testing (AST), particularly to colistin, to ensure treatment precision (Sacco et al., 2021). Accordingly, drug concentration monitoring and the accurate application of AST are indispensable when treating with colistin; in addition, the safety assessment of colistin monotherapy requires further study. Moreover, in treating the BSIs of carbapenem-resistant NFGNB, high-dosage tigecycline (TGC) therapy was not superior to standard TGC dosing, and TGC-based combination antimicrobial therapy was not superior to monotherapy (Qu et al., 2021). Thus, whether TGC is suitable for the treatment of CRAB infections requires further research and verification.

In addition to the treatment alternatives included in these guidelines, other treatment strategies have also been recently examined in response to the development of antibiotic resistance. Phage therapy is regarded as a promising selection for treating pulmonary bacterial infections. Wu et al. reported that using a pre-optimized two-phage cocktail in ICUs can cause a significant decrease in CRAB burden (Wu et al., 2021). Other treatment strategies in recent years also included N-acetylcysteine plus antibiotics (Oliva et al., 2021), trimethoprim–sulfamethoxazole (Raz-Pasteur et al., 2019), and other sulbactam-based combinations (Qu et al., 2020). These therapeutic strategies may be further studied in the future. However, integrated strategies for the effective prevention and control of infection caused by drug-resistant bacteria have to be urgently developed because of the evolution and spread of antibiotic resistance, as well as existing challenges and limitations in pharmaceutical research.

### Infection control strategies

Awareness of infection prevention and control has increased with intensified research in epidemiology. Agodi et al. indicated that infection control measures are equally important as the cautious use of antibiotics (Agodi et al., 2015). In 2017, WHO published the first global guidelines for the prevention and control of carbapenem-resistant *Enterobacteriaceae*–*A. baumannii*–*P. aeruginosa* in health care facilities, including eight evidence-based recommendations distilled by leading global experts (World Health Organization, 2017). Subsequently, the WHO global report on infection prevention and control provided a global situation analysis for policymakers at different levels, which can be used to facilitate the development of disease control strategies (World Health Organization, 2022). Accordingly, a growing number of healthcare workers have devoted themselves to the prevention and control of drug-resistant bacteria infections in ICUs. Thus, measures taken for CRAB infection control in ICUs are further discussed in the succeeding section.

#### Control of carbapenem use

The emergence, persistence, and dissemination of CRAB in ICUs limit therapeutic efficacy in critically ill patients. Antibiotic resistance is an ancient natural mechanism (Munita and Arias, 2016), but recent antibiotic usage effectively imposes selection pressure on ARGs. Selection pressure due to the use of antibiotics leads to the emergence, persistence, and dissemination of clinical resistant strains. Up to half of antibiotic courses might be inappropriate for use in ICUs (Barnes et al., 2017). Short-term carbapenem restriction effectively reduces the incidence of CRAB in ICUs (Ogutlu et al., 2014; Abdallah et al., 2019). Further, Munoz-Price et al. reported that every additive carbapenem-defined daily dose increased the risk of CRAB by 5.1% (Munoz-Price et al., 2016). Djordjevic et al. showed that previous use of carbapenems was a risk factor for CRAB infections in ICUs, and also demonstrated that appropriate policy of antibiotic utilization was an important measure that may decrease the incidence of such infections (Djordjevic et al., 2016). Therefore, controlling the usage of carbapenems to a certain degree can reduce the emergence and spread of CRAB in ICUs. However, the inevitable use of carbapenems prompts the need for stronger evidence to support and guide the use of antibiotics.

#### Early epidemiological screening for CRAB

As described previously (2.1), colonization by CRAB is a crucial step before nosocomial infection. Patients with CRAB intestinal colonization are more likely to develop CRAB infections, and admission screening of fecal carriage can prevent its spread (Maamar et al., 2018; Qiao et al., 2020). Culture processes and polymerase chain reaction (PCR) have long been regarded as the most common means of epidemiological screening for pathogens. Technological advancements have led to the development of new techniques to assist clinicians in early epidemiological screening for pathogens.

Investigators have detected *A. baumannii* and carbapenem resistance by loop-mediated isothermal amplification (LAMP) assay and found that LAMP was expected to act as an effective mean for early detection (Garciglia-Mercado et al., 2021; Sharma and Gaind, 2021). Rapid screening using LAMP assay followed by early intervention has also been reported to potentially decrease the transmission rates of CRAB in ICUs (Yamamoto et al., 2019). Li et al. optimized and evaluated the LAMP method, and their results showed that in *A. baumannii* detection, the sensitivity of LAMP was tenfold higher than that of PCR (Li et al., 2015). Garciglia-Mercado et al. reached a similar conclusion (Garciglia-Mercado et al., 2020). In addition to the LAMP assay, WGS was a valuable tool in epidemiological studies (Venditti et al., 2019). However, although the cost of sequencing has significantly decreased in recent years, WGS in epidemiological investigations remains extremely costly. Therefore, LAMP can potentially be used as an available screening method in epidemiologic investigations of CRAB, balancing sensitivity with the cost of detection.

#### Interventions

On the basis of the screening results, adequate control and disinfection measures can effectively limit the transmission of CRAB in ICUs. Chung et al. observed a 51.8% reduction in CRAB infection rates after daily chlorhexidine bathing in ICUs with CRAB endemicity (Chung et al., 2015). Other studies also indicated that daily bathing with 2% chlorhexidine gluconate can reduce CRAB cross-transmission among patients in ICUs with high CRAB endemicity (Hong et al., 2018; Metan et al., 2020; Suh et al., 2021). Moreover, environmental cleaning, isolation, and enhanced contact precautions are also integrated into strategies preventing CRAB cross-transmission among patients in ICUs because of the environmental reservoirs of CRAB.

Numerous studies have devoted themselves to preventing and addressing the environmental contamination and cross-transmission of CRAB in ICUs in recent years (Karampatakis et al., 2020; Dickstein et al., 2021; Jung et al., 2021; Kelly et al., 2021). First, the most significant point is that healthcare personnel education should be promoted to strengthen awareness and preparedness. Karampatakis et al. associated the increased infection rates for CRAB with work programs and behavioral factors (Karampatakis et al., 2020). Similarly, Kousouli et al. identified reduced compliance with hand hygiene and participation in educational courses as the most significant factor for CRAB bloodstream infection (Kousouli et al., 2018). A study in China found that after targeted surveillance, further implementation of infection control, including staff education, hand hygiene, and environmental cleaning can effectively prevent the spread of nosocomial CRAB infections (Zhao et al., 2019).

Second, new admissions should be separated from patients with colonization and infection, and their treatment should be handled by different medical staff members. An et al. isolated and grouped patients by CRAB culture results. They observed that the rate of CRAB infections and the use of colistin significantly decreased during the study period (An et al., 2017). Further, single-person isolation in ICUs was found to be an efficient method to prevent the transmission and hospital-acquired infections of CRAB (Guth et al., 2016; Jung et al., 2021).

Third, adequate environmental cleaning and disinfection also decrease the risk of transmission and infection of CRAB. Ben-Chetrit et al. showed that after environmental cleaning and hand hygiene, CRAB acquisition in ICUs considerably decreased from 54.6 to 1.9 (year 1) and 5.6 cases (year 2)/1,000 admissions (Ben-Chetrit et al., 2018). Various cleaning and disinfectant techniques have been widely used in health care settings to minimize HAIs. The use of phages as environmental sanitizers has been considered an alternative approach to removing bacterial contamination from the environment. Among the techniques reported, the use of phages as environmental sanitizers successfully decreased the rates of CRAB infection in ICUs (Ho et al., 2016; Chen et al., 2022). Steam technology (Oztoprak et al., 2019), terminal cleaning with sodium troclosene (Dickstein et al., 2021), installation of heat and moisture exchangers (Thatrimontrichai et al., 2020), ultraviolet-C, and aerosolized hydrogen peroxide (Kelly et al., 2021) have been successfully applied in environmental disinfection. By contrast, another study reported that strengthened environmental cleaning exhibited no association with the incidence (*p* = 0.156) and colonization pressure (*p* = 0.825) of CRAB in ICUs (Seok et al., 2021). The argument of whether decreasing the use of ventilators is more important than environmental cleaning was presented. A synthesis of the results of these studies indicates that controlling the use of ventilators for environmental disinfection may achieve significantly improved outcomes in infection control.

**Figure 3:**
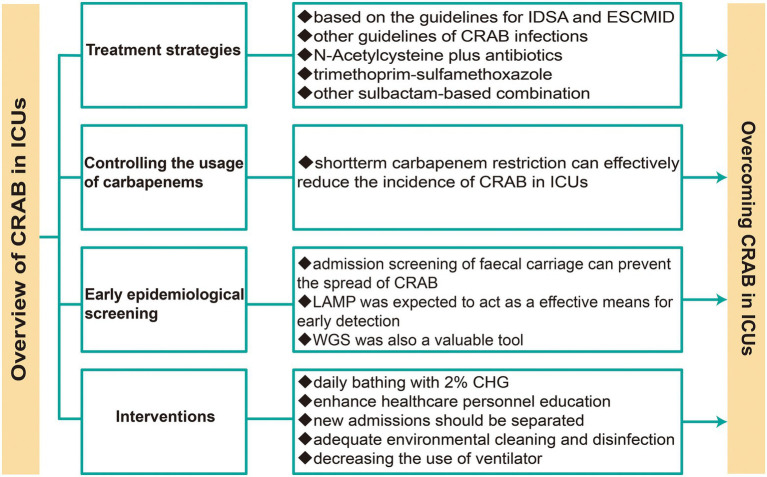
Treatment and infection control strategies of CRAB in ICUs.

Figure 3 recapitulates the treatment and infection control of CRAB in ICUs. Collectively, developing an integrated system to monitor the microbial profiles, usage of antibiotics, and resistance profiles in ICUs, as well as combining multiple interventions, is necessary for infection control of CRAB in ICUs.

## Conclusion

CRAB has progressed as a leading cause of HAIs worldwide, particularly in ICUs. Its spread and multiple-drug resistance considerably impedes the treatment of critically ill patients. On the basis of epidemiology and antibiotic resistance, the combined application of multiple interventions can effectively control the emergence and spread of CRAB, as well as provides hope for the control of CRAB infections in ICUs. Certainly, the implementation of control measures is of crucial importance and has to be extended to other wards for the eradication of CRAB from hospitals.

## Author contributions

All authors participated in drafting and revising the review, contributed to the article, and approved the submitted version.

## Funding

This study was supported by Sichuan Science and Technology Program (2022YFQ0093 and 2021YFH001).

## Conflict of interest

The authors declare that the research was conducted in the absence of any commercial or financial relationships that could be construed as a potential conflict of interest.

## Publisher’s note

All claims expressed in this article are solely those of the authors and do not necessarily represent those of their affiliated organizations, or those of the publisher, the editors and the reviewers. Any product that may be evaluated in this article, or claim that may be made by its manufacturer, is not guaranteed or endorsed by the publisher.
